# The veteran-centered care conferences: interprofessional education and community involvement facilitated by the health sciences librarian

**DOI:** 10.5195/jmla.2022.1491

**Published:** 2022-07-01

**Authors:** Karen S. Alcorn, Sarah K. McCord, Sheila M. Seed, Tammy Gravel, Amanda M. Morrill

**Affiliations:** 1 karen.alcorn@mcphs.edu, Reference and Instruction Librarian and Associate Professor, Division of Library and Learning Resources, Massachusetts College of Pharmacy and Health Sciences, Blais Library, Worcester, MA.; 2 sarah.mccord@mcphs.edu, Research Data and Informatics Librarian and Associate Professor, Division of Library and Learning Resources, Massachusetts College of Pharmacy and Health Sciences, Henrietta DeBenedictis Library, Boston, MA.; 3 sheila.seed@mcphs.edu, Professor and Chair of Pharmacy Practice, Massachusetts College of Pharmacy and Health Sciences, Worcester, MA.; 4 tammy.gravel@mcphs.edu, Dean, School of Nursing and Chief Nurse Administrator, Massachusetts College of Pharmacy and Health Sciences, Worcester, MA.; 5 amanda.morrill@mcphs.edu, Associate Professor of Pharmacy Practice, Massachusetts College of Pharmacy and Health Sciences, Manchester, NH.

**Keywords:** Veterans, veterans', health care, interprofessional education, case-based learning, story-based learning, interprofessional collaboration

## Abstract

**Background:**

Veterans have a variety of unique healthcare needs and receive care from both the US Department of Veterans Affairs (VA) and private healthcare systems. Because healthcare students will likely treat veterans at some time during their career, it is important they gain exposure to working with veterans during their professional degree programs.

**Case Presentation:**

This case report presents the development of an annual Veteran-Centered Care Conference (VCCC) at the Massachusetts College of Pharmacy and Health Sciences. The VCCC included a faculty librarian who led a multi-disciplinary team that planned and coordinated each event. Speakers and participants included university students and faculty from multiple healthcare disciplines, as well as representatives from the VA, veterans' advocacy groups, and community members (including many veterans). The purpose of the VCCC was to raise awareness of the healthcare needs of contemporary veterans. The goal of the VCCC was to improve healthcare provided to veterans by enhancing civilian health professions students' knowledge of the potential effects of military service on a person's health.

**Conclusion:**

After four successful events covering such topics as PTSD, specific health concerns of women veterans, substance use disorder, and homelessness, the VCCC was canceled, primarily due to low pre-registration. Examples of lessons learned and future possibilities for the VCCC and the patient-centered care conference format are discussed. This report is of particular importance given the many years the United States has been at war in the Middle East and the recent withdrawal of troops from Afghanistan.

## BACKGROUND

### Veterans and the US Healthcare System

There are an estimated 18.2 million veterans in the US, and many of these individuals have participated in multiple conflicts or wars [1]. These individuals receive care both within and outside of the Veteran Health System [2]. Regardless of the practice setting, clinicians may be challenged to complete applicable evidence-based assessments and develop appropriate treatment strategies for veterans who have a variety of unique healthcare needs, such as: traumatic brain injury, polytrauma, posttraumatic stress disorder (PTSD), military sexual trauma, chronic pain, and substance use disorders, among others [2].

Interprofessional collaboration, in which health professionals work collaboratively in interdisciplinary teams, can be used to address the needs of veterans and is a necessary component of comprehensive and safe patient care [3–4]. Interprofessional education (IPE) within university settings and healthcare agencies has been shown to contribute to successful collaborations across different health disciplines [5–6].

### An Opportunity for Collaboration

This case report takes place at the Massachusetts College of Pharmacy and Health Sciences (MCPHS), a multi-state health sciences university. MCPHS is made up of 12 Schools offering more than 100 academic programs to approximately 7,500 students, via three physical campuses as well as through online programs (see Table 1). MCPHS' primary focus is providing education across a variety of health profession disciplines, and faculty librarians are actively involved in achieving this mission as educators and collaborators.

**Table 1:** Massachusetts College of Pharmacy and Health Sciences University campus details.

MCPHS University Campus	Distance from Main Campus (miles)	Program Type
Boston, MA (Main Campus)	n/a	Traditional
Manchester, NH	56	Accelerated
Worcester, MA	47	Accelerated

A recent example of library-facilitated interprofessional and community collaboration was a series of events known as the Veteran-Centered Care Conference (VCCC). The VCCC took place in multiple formats, initially beginning at the Worcester campus and later growing to include participants and speakers from all campuses. The Worcester and Manchester campuses are home to accelerated programs for graduate degrees in nursing, occupational therapy, optometry, pharmacy, physical therapy, and physician assistant studies. After the first year, VCCC activities were conducted simultaneously at the Worcester and Manchester campus locations, using distance education (DE) technologies to connect participants with each other. Although the main Boston campus was included in one VCCC event, turnout was minimal and subsequent VCCC events did not include this campus.

During the summer of 2013, Johnson et al published an article entitled “Enhancing Veteran-Centered Care: A Guide for Nurses in Non-VA Settings” in the American Journal of Nursing [2]. The librarian shared this information with the School of Nursing faculty at the University. The librarian was invited to speak at a dean's meeting regarding this project, and the deans suggested that this topic be used in conjunction with the students' clinical rounds program, leading to a unanimous decision to plan an event in November around Veteran's Day. Each program identified a faculty member to join a task force to plan the event, and the librarian was charged with pulling the group together to plan and coordinate the event. It was suggested that the event be framed similarly to the Schwartz Rounds, a reflective practice which uses stories related to the emotional aspects of working in healthcare [7]. Story-based learning has been used successfully in healthcare settings, promoting engagement, and allowing participants to personally engage and reflect on a situation [8].

## CASE PRESENTATION

### Conference Planning and Logistics for First VCCC

Conference planning began with the formation of a committee and the appointment of the librarian and a nursing faculty member as co-chairs. Targeted emails were then sent out to multiple schools in hopes of soliciting faculty members who could identify student panelists for each topic. This step was crucial as the faculty were best able to approach students who were veterans or who had experience caring for veterans. Participants were recruited for both a student and a faculty/expert panel.

As prospective panelists were being identified, the planning committee grew to include members from different programs within the university who were excited to contribute to the conference. Four faculty members representing the Division of Library and Learning Resources, School of Nursing, and School of Pharmacy Practice made a commitment to serve as the core planning committee each year and founded the annual Veteran-Centered Care Conference.

As co-chair of the committee, the librarian took the role of principal investigator for the IRB protocol review and annual extensions and handled many of the key logistical duties for planning the conference. The librarian also scheduled early planning meetings, during which topic ideas were generated and potential speakers were identified. Speakers were identified using multiple means, including networking at conferences and reaching out to colleagues to solicit content ideas and contact information for potential speakers. Organizations with mission statements related to veteran health and social care were contacted directly to request their participation in the event.

Once the theme was chosen, the team created a save-the-date email, developed evaluation forms, and solidified arrangements for a moderator, professional expert panelists, and student panelists. The librarian took the lead with contacting the moderator and sending out emails soliciting faculty and student panel proposals, which included a form to help with the selection of panelists. As the designated contact person for the event, the librarian worked with students to fine-tune their presentations, coordinated travel and parking for speakers and guests, and was greeter and timekeeper on the day of the event. In addition, the librarian created all event materials, including the event agenda, biography handouts, a PowerPoint slideshow (which was shown prior to the event starting), and an IRB-approved survey of attendee experiences.

Following the event, the librarian wrote thank you notes and supporting letters for the panelists, compiled data from evaluations, and wrote a news blurb for the university newsletter and local media outlets. In later years, the librarian created and maintained a LibGuide to preserve information regarding the VCCC [9].

### Budget

Operating costs for these events were limited to the cost of refreshments and decorative items. There was no centralized source of funding for this conference. The first year the Office of the Provost covered the food costs; this was covered during the second year by the School of Nursing and the Division of Library and Learning Resources. No refreshments, other than bottled water donated by School programs, were provided in the third and fourth years. Across all four years, speakers were not compensated for their participation. All facility and technology costs for DE simulcasts were covered by the University as part of normal facilities operation.

### IRB Approval of Data Collection for Evaluation of VCCC

The University's Institutional Review Board approved a survey and the collection of data from VCCC participants (IRB072315L). Each year a four-question survey was distributed to participants immediately following the conference. Responses were used to ascertain the value of the conference to participants as well as the potential effects of the information gained on their future practice as healthcare providers.

Using a Likert scale (strongly agree, agree, neither agree or disagree, disagree, strongly disagree, not applicable) students were given a paper survey to voluntarily complete at the end of the session. Questions included participants' self-assessment of the following, as well as a space for comments:

- level of understanding of veterans' healthcare concerns,
- if VCCC experience will change the way they care for veterans in the future,
- if the material was presented in an acceptable format, and
- if they would choose to participate in future activities of this nature.

Student reflections were required from students who were receiving IPE credits for attending. A box on the form was available for students to check if they did not want their responses to be included in the research. The participants also had an opportunity to suggest topics for future conferences. Survey responses were collated, and aggregate data were shared with VCCC organizing committee members. The data were used to improve both the logistics and content delivery of the VCCC as well as develop future conference topics.

### The Veteran-Centered Care Conferences

Each VCCC was unique, but a universal format and flow was used each year. The core VCCC committee facilitated each event, and a keynote speaker opened the session, providing background information concerning the topic being addressed. All conferences were held in November near Veterans Day and included personalized stories from students, faculty, and expert panelists, many of whom were themselves veterans. These stories moved those in attendance and provided relevant perspectives regarding veterans' healthcare issues. Student panelists submitted a one-page story which was reviewed in advance by the librarian and another faculty member. During this review, the story was lightly edited to omit sensitive or unnecessary information to keep the story focused and able to be told in an approximate 5-minute timeframe. This format generates participant interest by engaging both their intellect and emotions and assisted our less-experienced panelists by providing them with a less formal and more comfortable way to share their experiences and knowledge.

All but the first conference incorporated an IPE “unfolding case” activity, created by the National League for Nursing [10], to foster audience participation and enhance student learning. Student participants were assigned to groups to ensure a mix of programs (e.g., Nursing, Pharmacy, Physician Assistant Studies, Physical Therapy, and Optometry) would be represented in each group. A member of the core VCCC committee, who was an experienced IPE instructor, facilitated this portion of the program. Attendees heard a case, reviewed a chart, and worked in IPE groups to collaborate on how they would treat their patient and answered questions provided about the case. The panelists then told their stories, sharing different perspectives and insight concerning the selected topics. Audience members then returned to their groups to review the unfolding case questions and discuss how their answers might have changed after listening to the panelists. Finally, attendees returned to the large group to share their various answers and viewpoints. See Table 2 for a summary of topics, participant areas, attendees, and format used during each conference.

**Table 2:** Summary of VCCC program details 2013-2016. (WOR = Worcester, MA; MAN = Manchester, NH; BOS = Boston, MA. Conference numbers are approximate, official counts were not taken.)

VCCC/Year	Topics relating to providing healthcare to veterans	Panelist/Speaker/Community/School/Specialty areas	Attendees	Format
1^st^ / 2013	Challenges of providing appropriate care; Impact of military service	Nursing, Physician Assistant, Pharmacy, Veterans, Creative Writing, Poet	~80	In-person (WOR), student and expert panels
2^nd^ / 2014	Post-traumatic stress disorder (PTSD); Impact of military service	Pharmacy, Physician Assistant, Poet, Veterans	~65	In-person (WOR) & DE (MAN), PTSD PPT presentation, PTSD unfolding case, student/expert panel
3^rd^ / 2015	Barriers to access; Unique challenges for women veterans; Caring for the veteran's family/care givers	Pharmacy, Poet, Braveminds, Physician Assistant, Nursing, Veterans, Optometry	~292	In person (WOR), DE (MAN & BOS) female veteran unfolding case, student/expert panel
4^th^ / 2016	Homelessne ss; Substance use disorder	Nursing, Optometry, Acupuncture, Physician Assistant, New England Center and Home for Veterans,	~230	In-person (WOR) & DE (MAN), homeless veteran unfolding case, student/expert panel

We used several methods to evaluate the VCCC, primarily relying on formal feedback from attendees using the IRB-approved questionnaire. Responses from all four VCCC events (2013-2016) are compiled and summarized in Table 3. The use of the IPE “unfolding case” activity in the second through fourth years of the conference prompted the development and use of an additional IPE reflection form for student participants interested in receiving IPE credit for participating in the conference.

**Table 3:** Compiled results of all Veteran-Centered Care Conference (VCCC) participant surveys, 2013-2016.

VCCC 2013–2016 Overall (n=502)	Strongly Agree 1	Agree 2	Neither Agree or Disagree 3	Disagree 4	Strongly Disagree 5	Not Applicable
This activity provided me with a better understanding of veterans' healthcare concerns	263 (52.4%)	207 (41.2%)	23 (4.6%)	5 (1.0%)	4 (0.8%)	
Attending this program will change the way I care for patients who identify as veterans	260 (51.8%)	181 (36.1%)	38 (7.6%)	14 (2.8%)	5 (1%)	4 (0.8%)
The format of this program was helpful in presenting multiple perspectives on healthcare challenges facing veterans	227 (45.2%)	199 (39.6%)	43 (8.6%)	23 (4.6%)	10 (2%)	
I would participate in more activities like this in the future	198 (39.4%)	190 (37.8%)	81 (16.1%)	17 (3.4%)	15 (3%)	1 (0.2%)

## DISCUSSION

### What Happened to the VCCC?

The fifth Annual VCCC was scheduled to take place at the Manchester campus. We did not plan to use DE for this conference because previous conferences were held at the Worcester campus with DE to the Manchester campus, and this was very unpopular with the Manchester students as indicated by their evaluation comments.

Topics of this conference were to be veteran suicide, access to healthcare, and substance use disorder. Scheduled panelists included students from the University, representatives from a veteran-centered charitable foundation, and a local VA pharmacist who represented an organization that helps individuals and their families live substance-free lives. Attendee comments from previous VCCCs requested panelists be given more time to speak because participants found the stories so meaningful. For the fifth VCCC, the organizers decided that each panelist would have 15 minutes to speak instead of 5 minutes. A recording of the event was also planned.

The conference was cancelled when only 16 students registered. This was not sufficient participation to provide a meaningful interprofessional education experience. The team felt that the limited free time of students in accelerated programs, the absence of mandatory attendance requirements, and the choice of hosting the event on the smallest campus location all contributed to the low registration and ultimate cancellation of the event. The idea of a spring conference was briefly entertained, but never pursued for the same reasons that resulted in the cancellation of the originally scheduled conference.

### Lessons Learned

There are several takeaways from our experiences for librarians considering future use of this model, most of which can be summarized as issues related to technical or logistical challenges, or the need for soliciting additional institutional support. The major technical challenge noted on evaluation forms was student dissatisfaction with DE technology. Poor-quality audio and inexpert camera work made it difficult for some participants to hear, see, and feel connected to the speakers, reducing the impact of the storytelling portion of the conference.

Logistically, an official registration process was not required for participants and was not an issue until the third year when the attendance spiked to almost 300, leading to cramped accommodations and the need to turn people away at the door. The fourth conference required registration and those not registered on the date of the event were admitted on a first-come first-served space-available basis just before the conference started. Another issue was the evaluation survey we used. Some of the responses were inconsistent with comments made on the evaluations. One participant marked all the “strongly disagree” categories but had nothing but praise and positive comments for the program on the same form. When it was investigated, it was learned that student evaluations for credit-bearing courses, given at the end of each semester by our institution, used the same scale, but had the “strongly agree” in the same location that we had our “strongly disagree”. The accelerated nature of the programs on the Worcester and Manchester campuses may have also presented a logistical issue. These programs feature full daily schedules, limiting the availability of faculty and students' participation in extracurricular activities. This event may be more successful on a campus with traditional programs, where students have more flexibility to participate.

Areas in which it would be beneficial to solicit official institutional support include the IPE portion of our event. Although the VCCC program had an IPE component, it was not formally endorsed by the University's IPE Working Group and the thoughtful student reflections on the interactive IPE portion of the program were never formally analyzed. Student participation in the VCCC was not consistently made mandatory for students in most programs, resulting in difficulties creating groups that included a mix of healthcare disciplines. Additional collaboration with faculty members to secure dedicated class time to the VCCC may have helped sustain it. In general, student responses to the IPE portion of the event indicated they felt it was worthwhile. However, some participants felt the “unfolding case” activity did not flow well with the rest of the conference content and would have preferred more time devoted to the panelists' stories.

Obtaining a dedicated budget to provide food and beverages may also have increased registration for the VCCC, allowing it to continue beyond the initial four years. The conference was several hours long and often lasted into the noon hour, and many students commented on the lack of refreshments. Providing lunch during the event may have increased the appeal of the program for students with tight schedules.

Not all the lessons learned were about challenges or barriers. Participants consistently praised the story-based learning component and requested that more time be devoted to the panelists. A veteran poet and master storyteller who participated in the first three VCCCs captivated the audience, and attendees praised his contributions. Most evaluations from all four years showed a favorable response to this program. One student sent an email to the lead author thanking her for hosting this program and stating that they were going to dedicate their career to serving veterans once they graduated. The open-response questions on the survey form further demonstrated the impact of the VCCC on student perceptions and practice, as indicated through comments such as:

“I now have a better understanding of what our veterans go through.”

“It was an honor to listen to these stories, and it will affect my care as a health care professional.”

“It added information and reinforced some key points in my knowledge base.”

The future of the VCCC at this institution remains uncertain. Over the four years of the VCCC, the core team members all moved into positions which required more of their time, resulting in less availability for staging this event. The uncertainty surrounding the COVID-19 pandemic has also had an impact on any progress in reinstating this conference.

### Future Plans/Moving Forward

Providing appropriate and sensitive care to veterans remains an issue in the US healthcare system, and the need for healthcare education in this area is still great. At the same time, the increased use of technology in remote learning and working due to the global pandemic has provided new opportunities for expanded attendance and participation in events like the VCCC [11]. Participants and panelists now have both more experience with and many more models for how to interact using general technologies such as Zoom or integrated online tools available through learning management systems [11–12]. This could be a way to address the significant participant dissatisfaction with the DE technologies that were available between 2013-2017.

Because of an overall increase in familiarity and comfort with tools like Zoom, organizers of future conferences could make use of pre-recorded or asynchronous content that would better meet the time constraints experienced by students in accelerated programs. Pre-recorded content and virtual access to the VCCC could also improve access for students at all three campuses or enrolled in online programs, would allow instructors to assign VCCC content to students as a required part of coursework, and could also increase the number of stories available for students to experience and learn from [12]. Conference organizers could potentially use these tools to allow veteran and expert panelists to participate from distant locations, which could also provide benefit to the veteran participants by providing them with a respectful, safe, and validating interaction with health care professionals.

The VCCC may have had its challenges, yet it remains a worthwhile event that can inspire students to be mindful of the unique needs of veterans when they serve members of this population. In addition to the veteran cases, the National League for Nursing has created other Advanced Care Excellence (ACE) cases for other patient populations, including children, older adults, and persons with disabilities [10]. The focus of the conference could be adapted to use these “unfolding cases” for other groups of patients in conjunction with story-based learning to provide participants with a rich IPE experience. Medical librarians can use the VCCC format as a blueprint to develop these and other patient-centered care events, with the goal of making a difference in how healthcare students learn to care for diverse patient populations.

## Data Availability

Summarized yearly data associated with this article is available as an appendix to this article.

## SUPPLEMENTAL FILES

**Appendix A:** Program Evaluation Form

**Appendix B:** Student Reflection Form

**Appendix C:** Summarized Yearly Evaluation Data
