# Supplementary material for: The veteran-centered care conferences: interprofessional education and community involvement facilitated by the health sciences librarian
Source: J Med Libr Assoc. 2022 Jul 1;110(3):365–71. doi: 10.5195/jmla.2022.1491 (PMC9782503; doi:10.5195/jmla.2022.1491)
Supplement: Supplementary file 1 — Appendix A: Program Evaluation Form [file jmla-110-3-365-s01.pdf]

Internal use

Veteran-Centered Care Conference

Program Evaluation

You are being asked to participate in a research project. Research is a way of getting new knowledge. The purpose of this research project is to learn about student perceptions of the effectiveness of an interprofessional program to promote awareness and understanding of veteran health care issues. If you choose to participate, please complete the survey below. Completing the survey will take approximately 5 minutes.

You do not need to participate in this research. There is no penalty for deciding not to participate. No one will know who participated and who did not.

If you have any questions about the research, please contact the Principal Investigator, [redacted], at [redacted]. If you have any questions about your rights as a participant in research, please contact [redacted], Chair of the [redacted] Institutional Review Board at [redacted].

Please indicate which best describes your role here today: Student Faculty Healthcare provider Other

	StronglyAgree 1	Agree 2	Neither Agree or Disagree 3	Disagree 4	Strongly Disagree 5	Not Applicable
This activity provided me with a better understanding of veterans' healthcare concerns						
Attending this program will change the way I care for patients who identify as veterans						
The format of this program was helpful in presenting multiple perspectives on healthcare challenges facing veterans						
I would participate in more activities like this in the future						

Please provide suggestions for future events such as this:

Additional Comments:
