# Supplementary material for: The veteran-centered care conferences: interprofessional education and community involvement facilitated by the health sciences librarian
Source: J Med Libr Assoc. 2022 Jul 1;110(3):365–71. doi: 10.5195/jmla.2022.1491 (PMC9782503; doi:10.5195/jmla.2022.1491)
Supplement: Supplementary file 2 — Appendix B: Student Reflection Form [file jmla-110-3-365-s02.pdf]

Internal use

Veteran-Centered Care Conference

Student Reflection

You are being asked to participate in a research project. Research is a way of getting new knowledge. The purpose of this research project is to learn about student perceptions of the effectiveness of an interprofessional program to promote awareness and understanding of veteran health care issues. If you choose to participate, please complete the survey below. Completing the survey will take approximately 5 minutes.

You do not need to participate in this research. There is no penalty for deciding not to participate. No one will know who participated and who did not.

☐ Check this box if you DO NOT want your responses to be included in the research.

If you have any questions about the research, please contact the Principal Investigator, [redacted], at [redacted]. If you have any questions about your rights as a participant in research, please contact [redacted], Chair of the [redacted] Institutional Review Board at [redacted].

Directions: Please provide a short paragraph answering the following questions and submit to a facilitator before leaving the conference today.

Name: ____

School: ____

1. What did you learn from today's session and why is it meaningful to you?

2. How will you apply what you learned today in professional interactions with other disciplines in your clinical practice setting?

3. Explain a scenario described today when a provider or student from another discipline had a perspective different from yours and how that affected your perspective on the situation.
