# Supplementary material for: The veteran-centered care conferences: interprofessional education and community involvement facilitated by the health sciences librarian
Source: J Med Libr Assoc. 2022 Jul 1;110(3):365–71. doi: 10.5195/jmla.2022.1491 (PMC9782503; doi:10.5195/jmla.2022.1491)
Supplement: Supplementary file 3 — Appendix C: Summarized Yearly Evaluation Data [file jmla-110-3-365-s03.pdf]

Appendix C: Summarized Yearly Evaluation Data

1st Annual VCCC – 2013<br>Worcester Campus (n=62)	Strongly Agree<br>1	Agree<br>2	Neither agree or disagree<br>3	Disagree<br>4	Strongly Disagree<br>5	Not Applicable
This activity provided me with a better understanding of veterans' healthcare concerns	28<br>45.2%	32<br>51.6%	2<br>3.2%	0<br>0.0%	0<br>0.0%	0<br>0.0%
Attending this program will change the way I care for patients who identify as veterans	26<br>41.9%	30<br>48.4%	2<br>3.2%	2<br>3.2%	1<br>1.6%	1<br>1.6%
The format of this program was helpful in presenting multiple perspectives on healthcare challenges facing	24<br>38.7%	30<br>48.4%	6<br>9.7%	2<br>3.2%	0<br>0	0<br>0.0%
I would participate in more activities like this in the future	17<br>27.4%	28<br>45.2%	10<br>16.1%	4<br>6.5%	3<br>4.8%	0<br>0.0%

2nd Annual VCCC – 2014<br>Worcester/Manchester Campuses (n=49)	Strongly Agree<br>1	Agree<br>2	Neither agree or disagree<br>3	Disagree<br>4	Strongly Disagree<br>5	Not Applicable
This activity provided me with a better understanding of veterans' healthcare concerns	41<br>83.7%	7<br>14.3%	0<br>0.0%	0<br>0.0%	1<br>2.0%	0<br>0.0%
Attending this program will change the way I care for patients who identify as veterans	40<br>81.6%	7<br>14.3%	1<br>2.0%	0<br>0.0%	1<br>2.0%	0<br>0.0%
The format of this program was helpful in presenting multiple perspectives on healthcare challenges facing	37<br>75.5%	10<br>20.4%	1<br>2.0%	0<br>0.0%	1<br>2.0%	0<br>0.0%
I would participate in more activities like this in the future	35<br>71.4%	13<br>26.5%	0<br>0.0%	0<br>0.0%	1<br>2.0%	0<br>0.0%

3rd Annual VCCC – 2015<br>Worcester/Manchester/Boston Campuses (n=203)	Strongly Agree<br>1	Agree<br>2	Neither agree or disagree<br>3	Disagree<br>4	Strongly Disagree<br>5	Not Applicable
This activity provided me with a better understanding of veterans' healthcare concerns	122<br>60.1%	73<br>36.0%	6<br>3.0%	0<br>0.0%	2<br>1.0%	0<br>0.0%
Attending this program will change the way I care for patients who identify as veterans	121<br>59.6%	68<br>33.5%	7<br>3.4%	4<br>2.0%	1<br>0.5%	2<br>1.0%
The format of this program was helpful in presenting multiple perspectives on healthcare challenges facing	105<br>51.7%	73<br>36.0%	15<br>7.4%	6<br>3.0%	4<br>2.0%	0<br>0.0%
I would participate in more activities like this in the future	95<br>46.8%	71<br>35.0%	26<br>12.8%	6<br>3.0%	5<br>2.5%	0<br>0.0%

4th Annual VCCC – 2016<br>Worcester/Manchester Campuses (n=188)	Strongly Agree<br>1	Agree<br>2	Neither agree or disagree<br>3	Disagree<br>4	Strongly Disagree<br>5	Not Applicable
This activity provided me with a better understanding of veterans' healthcare concerns	72<br>38.3%	95<br>50.5%	15<br>8.0%	5<br>2.7%	1<br>0.5%	0<br>0.0%
Attending this program will change the way I care for patients who identify as veterans	73<br>38.8%	76<br>40.4%	28<br>14.9%	8<br>4.3%	2<br>1.1%	1<br>0.5%
The format of this program was helpful in presenting multiple perspectives on healthcare challenges facing	61<br>32.4%	86<br>45.7%	21<br>11.2%	15<br>8.0%	5<br>2.7%	0<br>0.0%

I would participate in more activities like this in the future	51<br>27.1%	78<br>41.5%	45<br>23.9%	7<br>3.7%	6<br>3.2%	1<br>0.5%

502

Summary Statistics All Responses VCCC – 2013-2016 n=502	Strongly Agree 1	Agree 2	Neither agree or disagree 3	Disagree 4	Strongly Disagree 5	Not Applicable
This activity provided me with a better understanding of veterans' healthcare concerns	263<br>52.4%	207<br>41.2%	23<br>4.6%	5<br>1.0%	4<br>0.8%	0<br>0.0%
Attending this program will change the way I care for patients who identify as veterans	260<br>51.8%	181<br>36.1%	38<br>7.6%	14<br>2.8%	5<br>1.0%	4<br>0.8%
The format of this program was helpful in presenting multiple perspectives on healthcare challenges facing	227<br>45.2%	199<br>39.6%	43<br>8.6%	23<br>4.6%	10<br>2.0%	0<br>0.0%
I would participate in more activities like this in the future	198<br>39.4%	190<br>37.8%	81<br>16.1%	17<br>3.4%	15<br>3.0%	1<br>0.2%
